# Minimizing the Silver Free Ion Content in Starch Coated Silver Nanoparticle Suspensions with Exchange Cationic Resins

**DOI:** 10.3390/nano12040644

**Published:** 2022-02-15

**Authors:** Catarina S. M. Martins, Alberto N. Araújo, Luís Pleno de Gouveia, João A. V. Prior

**Affiliations:** 1 LAQV, REQUIMTE, Laboratory of Applied Chemistry, Department of Chemical Sciences, Faculty of Pharmacy, University of Porto, 4050-313 Porto, Portugal; 2 Pharmacological and Regulatory Sciences Group (PharmaRegSci), Research Institute for Medicines (iMed.ULisboa), Faculdade de Farmácia da Universidade de Lisboa, 1649-003 Lisbon, Portugal

**Keywords:** silver nanoparticles, AgNPs, purification, separation, resin, cation exchange, green chemistry

## Abstract

This work describes the optimization of a methodology for the reduction of silver ions from silver nanoparticle suspensions obtained from low-yield laboratory procedures. The laboratory synthesis of silver nanoparticles following a bottom-up approach starting from silver nitrate, originates silver ions that were not reduced to their fundamental state for nanoparticles creation at the end of the process. However, it is well known that silver ions can easily influence chemical assays due to their chemical reactivity properties and can limit biological assays since they interfere with several biological processes, namely intracellular ones, leading to the death of living cells or organisms. As such, the presence of silver ions is highly undesirable when conducting biological assays to evaluate the influence of silver nanoparticles. We report the development of an easy, low-cost, and rapid methodology that is based on cation exchange resins to minimize the silver ion content in a raw suspension of silver nanoparticles while preserving the integrity of the nanomaterials. This procedure preserves the physical-chemical properties of the nanoparticles, thus allowing the purified nanoparticulate systems to be biologically tested. Different types of cationic resins were tested, and the developed methodology was optimized by changing several parameters. A reduction from 92% to 10% of free silver/total silver ratio was achieved when using the Bio-Rad 50W-X8 100–200 mesh resin and a contact time of 15 min. Filtration by vacuum was used to separate the used resin from the nanoparticles suspension, allowing it to be further reused, as well as the purified AgNPs suspension.

## 1. Introduction

From wastewater treatment to textile products, from food supplements to cosmetics, and from pharmaceutical products to food packaging materials, silver nanoparticles (AgNPs) are widely used for their properties, mainly their peculiar interaction with light and their potential to interfere with vital cellular processes [1]. Unfortunately, this gives rise to an increase in the levels of these nanoparticles in the environment, which can cause concomitant-related problems to human health. In recent years, different strategies have been studied and improved to reduce the dissemination of AgNPs in the environment. However, it is worth mentioning that many methodologies for the separation and/or purification of silver nanoparticles (AgNPs) from different matrices are in development, many of which are based on magnetic based schemes, chromatography, centrifugation, electrophoresis, selective precipitation, and extraction [2,3]. But, these methods are often used for Ag speciation and thus imply AgNPs disintegration for quantification measurements, making their posterior use unfeasible.

Considering separation by centrifugation, sucrose density gradient centrifugation is typically used [4,5]. The separation of metal nanoparticles from aqueous suspensions remains an issue since gradient centrifugation methods require a straight interval between the densities of the particles and the separation media, which cannot be fulfilled when separating metal nanoparticles, which usually have densities superior to the commercially available gradient media (1.4 g cm^–3^) [6]. This classical low-cost technique was recently used by Asnaashari Kahnouji et al. [4] to separate AgNPs from newly prepared suspensions, with particle sizes ranging from 15 to 235 nm. The authors achieved the fractionation of AgNPs with different-sized layers, and the AgNPs were kept intact to be used in further characterization studies.

Some authors have applied magnetic-based schemes to capture AgNPs from their medium through the adsorption of the AgNPs on magnetic nanoparticles [7,8] or by exploiting the incorporation of AgNPs in magnetic nanocomposites, such as Fe_3_O_4_-Ag(0) [9]. In the work of Mwilu et al. [7], the analytical procedure allowed the AgNPs to be preconcentrated by selective separation from the ionic silver (Ag^+^) present in the bulk solution and were thereafter quantified by ICP-MS. Yet, these methods are dependent on the coupling of AgNPs with a magnetic material, which might be cumbersome if one intends to use isolated AgNPs for other assays.

Alternatively, it is possible to reuse AgNPs through the use of Field–Flow Fractionation (FFF) [10]. Asymmetrical flow FFF (AF4) is the most well-known variant of this method [2]. With AF4, the AgNPs are separated inside a narrow channel and are eluted according to their size, due to a balance between the laminar flow forces and an applied perpendicular force. Among other works found in the literature [11,12,13,14,15], Ramos et al. [16] used AF4 to separate AgNPs from Korean beer and from commercial nutraceutical products. However, this approach requires high-end equipment, specialized personnel, and time. Additionally, the interaction of the AgNPs with the separating membranes leads to low rates of recovery.

Many chromatographic variants can be used for AgNPs separation [17,18,19,20,21], and the one that stands out the most is size exclusion chromatography (SEC) [22,23,24]. Zhou et al. [23] applied the SEC method in antibacterial products and environmental water samples to separate soluble Ag(I) species from the AgNPs that were present. Nonetheless, this technique has several disadvantages: (i) resolution depends on the differences in the molecular masses of the nanoparticles, which can be so small that it renders the SEC procedure unviable; (ii) possible interaction between the stationary phase and the nanoparticles, leading to longer elution times and, consequently, erroneous size population results; (iii) poor selectivity; and (iv) limited loading capacities of the samples.

Separation by means of capillary electrophoresis (CE) is one of the best known and most used methods [25,26,27,28]. Liu et al. [29] combined CE with a diode array detection system in order to simultaneously purify and characterize AgNPs. Agarose gel electrophoresis is another common technique among electrophoretic methods. For example, through agarose gel electrophoresis, Hanauer et al. [30] separated AgNPs according to their size and shape. However, these methods are highly dependent on specific nanoparticle characteristics and are often affected by problems deriving from capillary heating nanoparticle instability in separation conditions, which can lead to aggregation phenomena and capillary clogging, and thus, a lack of robust and reproducible separations [31].

Regarding the methods that can be used for the separation of AgNPs through selective precipitation, the gas-expanded liquid (GEL) approach [32], such as through the use of the CO_2_-expanded liquid developed by McLeod et al. [33], allows for the selective precipitation of AgNPs. In a gas-expanded liquid, the mixture of an organic solvent with a compressible gas (such as CO_2_ or ethane) the properties are different from those at atmospheric pressure. By increasing the pressure of the gas in a closed container, the viscosity of the GEL changes, and AgNPs of different size ranges precipitate, with the larger ones precipitating first, and then the smaller ones.

Beyond this approach, other methods can be used, such as the addition of a non-solvent that does not allow the NPs to solubilize, through the addition of salts, or through the addition of a supercritical fluid that acts as a solvent with density-tunable dissolving power [34,35].

Cloud-Point extraction (CPE) can be a method of choice for the separation and purification of AgNPs. It allows the preconcentration of silver as well as other trace elements from environmental and biological samples. In fact, for the first time, Yu et al. [36] proceeded to the separation of AgNPs and Ag^+^ in HepG2 cells lysates using Triton-X 114-based CPE. Other examples of the use of CPE to preconcentrate silver and AgNPs in environmental waters can be found in the literature [37,38,39], but a list of limitations is thoroughly discussed in the work of S. S. Arya et al. [40].

Whenever AgNPs are used in cellular assays, the presence of Ag^+^ and other waste materials resulting from their synthesis is a concern. The Ag^+^ can introduce bias into study outcomes since the obtained results may not be dependent on the silver that is present only in the nanoparticle form, but rather on the total silver present in the medium (free Ag^+^ inclusive). This aspect is of paramount importance when green-based synthesis procedures are used to obtain AgNPs since these methods often present low synthesis yields because of the weaker reducing agents when applied for the formation of the silver nanoparticles, but they are greener. In the present work, the application of ion-exchange resins (IER) to separate free silver ions from synthesized AgNPs is described, enabling studies with purified AgNPs and cells or studies that require the absence of cytotoxicity by free Ag ions. Most IER are synthesized from the polymerization of chemicals deriving from petroleum, namely acrylic acid, styrene, and divinylbenzene, among others [41]. The mechanism of action of the IER between the charged ions is based on positive ion exchange if the resin is cationic or on negative exchange if the resin is anionic [41], and the ion exchange process takes place inside the porous media of the resin [42]. Basically, in an aqueous medium, the dissolved ions are removed through replacement with other ions. An example of a cationic exchange would be sodium for calcium, and an example of an anionic exchange would be chloride for arsenic because the resin beads attract ions of an opposite charge [43]. After being used, the IER can be recovered because they can be separated by filtration and then regenerated, making IER-based separation methods ecologically cleaner [44]. The literature contains some works exploiting cationic resins to separate Ag^+^ from samples containing AgNPs [45,46], but these are often focused on Ag^+^ quantification rather than on the purification of AgNPs. In the work of Iglesias et al. [45], the authors used IER to eliminate the interference of Ag^+^ in the SP-ICPMS measurements, but humic acid was added to stabilize commercial PVP and citrate-AgNPs [47]. Another work [48] exploited AgNPs adsorption onto an anionic exchange resin, Amberlite IRN-78, after the chemical modification of the AgNPs surface with mercaptosuccinic acid that had been negatively charged in a basic medium.

This work describes the optimization of a methodology based on a cationic exchange resin for the separation of Ag^+^ from the AgNPs suspensions after their synthesis in a single extraction procedure and aims to achieve the highest purification ratio of starch-stabilized AgNPs suspensions synthetized in a laboratory. By following a green-based synthesis approach, using starch and glucose to produce AgNPs, yields of around 8% were obtained, and these were the target suspensions for the purification procedure. Upon characterization studies, it was verified that the resultant purified AgNPs remained intact after the procedure, enabling their further use, and they were largely free from the interference of silver ions.

## 2. Materials and Methods

### 2.1. Reagents and Equipment

All solutions were prepared with deionized water obtained by a water purification system from Heal Force, model Easy, with ASTM Type I ultra-pure water provided (conductivity ≤ 0.1 μs cm^–1^). The reagents, potato starch soluble (Merck, Darmstadt, Germany), anhydrous D-glucose (Fisher Scientific, Loughborough, UK), and silver nitrate (LabKem, Barcelona, Spain), were of analytical grade and were used without any further purification. The strongly acidic resin Dowex 50W-X8, 50–100 mesh was acquired in its hydrogen form from Supelco (Sigma Aldrich, Madrid, Spain). The cation exchange resins Bio-Rad AG^®^ 50W-X8, 100–200 mesh and 200–400 mesh, both in their hydrogen forms, were purchased from Bio-Rad Laboratories (Bio-Rad Laboratories Inc., Algés, Portugal).

Two solutions, HNO_3_ 20% and 5% (*v*/*v*), were prepared from concentrated HNO_3_ (Fluka^TM^, Seelze, Germany). Finally, pure acetone and a silver standard solution (1000 mg/L) were purchased from Prolabo^®^ (Bois, France) and from SCP Science (Villebon-sur-Yvette, France), respectively.

Silver nitrate (AgNO_3_) 0.1 mol L^–1^ and glucose (C_6_H_12_O_6_) 0.07 mol L^–1^ stock solutions were prepared daily by the direct dissolution of the corresponding weighed masses of the reagents in deionized water.

The potato starch [(C_6_H_10_O_5_)_n_] stock solution, which had a fixed concentration of 0.65% (*w*/*v*), was daily prepared in a microwave reaction vessel (35 mL) by weighing the calculated mass of the starch, adding the necessary volume of deionized water, and dissolving it with the aid of microwave heating under continuous magnetic stirring at 95 °C for 270 s. In the end, the prepared starch stock solution was transferred to an amber glass flask and cooled at room temperature before further use.

A CEM Discover SP^®^ microwave synthesizer (CEM Corporation, Matthews, NC, USA) operating with the Synergy^TM^ software was used to prepare the starch solutions and for the syntheses of the AgNPs.

A double beam spectrophotometer Jasco V-660 (Jasco, Easton, MD, USA) was used to characterize the AgNPs in UV-Vis. The TEM analysis was performed using a JEOL 2100 electron microscope (JEOL Ltd., Tokyo, Japan) operating at 200 kV with a high brightness LaB6 electron gun equipped with a fast-readout “OneView” 4 k × 4 k CCD camera.

Silver quantification was performed by atomic absorption spectrometry using a Perkin Elmer AAnalyst 200 spectrometer (Perkin Elmer, Waltham, MA, USA) equipped with a 50 mm lamp model HCL-AG-50-A from Kinesis Ltd. (Cambridgeshire, UK). An Allegra^®^ X-15R centrifuge (Beckman Coulter, Pasadena, CA, USA) was used when necessary.

### 2.2. Synthesis of Starch Stabilized Silver Nanoparticles

**Figure 1:**
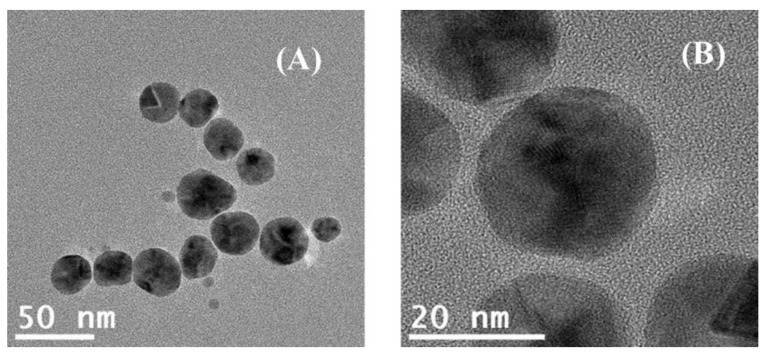
TEM images of AgNPs at different magnifications: (**A**): 50 nm; (**B**): 20 nm.

The starch-stabilized AgNPs (starch–AgNPs) were synthesized using a green chemistry approach [49] in a microwave synthesizer to ensure the rigorous control of the irradiation time, the reaction temperature, pressure, power, and stirring. In an aqueous medium, AgNO_3_ was used as a precursor, glucose was used as a reductant, and starch was used as a coating and stabilizing agent for the nanoparticles. Glucose is a reducing sugar and contains a linear aldehyde function that can be readily oxidized to carboxylic acid under the right conditions. Starch is mostly composed of glucose units that are linked together and has diverse degrees of branching as well as amylopectin and amylose contents. It is a low-cost reagent, easily accessible, environmentally friendly, and ensures the effective stabilization of the as-synthesized nanoparticles [50]. In a 35 mL reaction vessel, pre-determined volumes of ultrapure water, starch solution, D-glucose stock solution, and AgNO_3_ stock solution were added in order, resulting in a total volume of 22 mL and aiming at the following reagent ratios in the reaction vessel: Ag/starch 1.0:1.3 (mass ratio) and Ag/glucose 1.0:1.5 (molar ratio). The synthesis was performed with automatic stirring set at High, the heating temperature set at 158 °C, a 270 s of heating time, and a microwave power fixed at 300 W. In the end, a yellow transparent suspension was obtained due to the formation of silver nanoparticles. Besides the UV-Vis spectral analyses, a small aliquot was dripped onto a 400-mesh carbon copper grid (Ted Pella, Inc., Redding, CA, USA) followed by drying over filter paper in order to conduct TEM analysis (Figure 1).

### 2.3. Quantification of AgNPs and Free Ag^+^

For the determination of the suspended AgNPs concentration by AAS, the nanoparticles contained in 1 mL aliquot were precipitated with the addition of 1 mL of pure acetone followed by vigorous vortex mixing for 90 s and centrifuging for 9 min at 2500 RCF at room temperature. Then, the supernatant was separated, and the pellet was resuspended in 1 mL of ultrapure water. The separated supernatant provided the free Ag^+^ concentration that had not been reduced to silver nanoparticles. On the other hand, the determination of the silver content in the resuspended pellet provided the Ag concentration in the form of AgNPs. As a result, the AgNPs concentration is expressed as the Ag content. Before the AAS measurements, all of the samples were properly diluted using HNO_3_ 5% (*v*/*v*) in order to fit the analytical signals in the linear interval being used for calibration, which was between 1 and 4 mg/L.

### 2.4. Assays with Cationic Exchange Resins

Different cationic exchange resins (CER) were used to recover the ionic silver from the starch-AgNPs suspensions produced in the laboratory, namely the Dowex 50W-X8 50–100 mesh, Bio-Rad 50W-X8 100–200 mesh, and Bio-Rad 50W-X8 200–400 mesh (Table 1) suspensions. Each resin was regenerated through stirring in HNO_3_ 20% (*v*/*v*) for 1 h at room temperature, then filtered under vacuum, rinsed several times with ultrapure water, and finally oven-dried at 60 °C.

In order to evaluate the resin performance, 20 mL of a starch-AgNPs suspension was added to a beaker containing a fixed amount of resin, where it stayed for a total time of 90 min, during which some supernatant aliquots (2.0 mL) were extracted at pre-fixed time intervals for the posterior UV-Vis and AAS analyses. The influence of mechanic stirring on the separation process was also evaluated. All experiments were conducted in triplicate.

To simplify the evaluation of the results, the purification ratio (%) was used to express the amount of silver in the form of AgNPs relative to the total amount of silver in the suspension (Ag^+^ plus AgNPs) using the formula: purification ratio (%) = $\frac{\;C_{t}}{Ct_{t}}\times 100$, where *C_t_* is the AgNPs concentration at a determined contact time *t* with the resin, and *Ct_t_* is the total Ag concentration measured in the suspension at the same time *t*. To determine the concentration *Ct_t_*, the samples were acidified with nitric acid and were directly analyzed by AAS without a pre-precipitation treatment, whilst the *Ct* values (AgNPs content) were obtained after precipitation, and the pellet was separated as already previously described in Section 2.3. Using the same results from the AAS measurements, the AgNPs removal ratio (AgNPs ratio) was evaluated by the formula $\frac{\;C_{t}}{Ct_{0}}$. In this equation, *C_t_* is AgNPs concentration at a determined contact time *t* with the resin, and *Ct_0_* is the total Ag concentration measured in the suspension at 0 min. The removal ratio of the total silver content (Ag Total ratio) from the suspensions was evaluated by applying the formula $\frac{\;Ct_{t}}{Ct_{0}}$, in which *Ct_t_* was the total Ag concentration measured in the suspension at the same time *t,* and *Ct*_0_ was the total Ag concentration measured in the suspension at 0 min.

## 3. Results and Discussion

### 3.1. Preliminary Assays

Assays were conducted to first evaluate the potential of using cationic exchange resins to separate silver ions from the AgNPs suspensions obtained through chemical synthesis. These assays consisted of testing two different masses of the resin Dowex 50W-X8 50–100 mesh (0.50 and 1.00 g). First, Ag (Total) and Ag (AgNPs) concentrations in the raw suspension were determined, and afterwards, an aliquot of 10 mL suspension was put in contact with a weighed mass of resin, which was manually shaken for 5 s at 20 min intervals. At the 1 h and 2 h contact times, 1.5 mL of supernatant was removed to determine the Ag (total) and Ag (AgNPs) again. The obtained results are depicted in Table 2.

Considering the difference in the C_Ag total_ values at time 0 and 1 h interval, the obtained results showed a significant reduction in the free silver ion content due to the cationic ion exchange processes, demonstrating the potential usefulness of the resin. The amount of AgNPs (Ag in AgNPs) also decreased with the contact time, revealing unwanted losses in the process. It is possible that a small portion of the AgNPs was adsorbed by the resin or was disintegrated during the purification process. The calculated purification ratio (%) at 0 and 1 h were 5.69% and 43.1%, respectively, for the resin mass of 0.50, and after 2 h of contact, the AgNPs % ratio increased to 88.5%. Similar observations were observed when using 1.00 g of the resin. Overall, the obtained results in the preliminary assays confirmed that despite the AgNPs losses during the adsorption process, the application of the resins to purify the AgNPs suspensions enabled purer suspensions to be obtained without a significant amount of silver ions and with CER selectivity towards the free silver ions.

In order to reach optimal performance for the proposed methodology, the effect of several factors in the purification ratio were evaluated, namely the (i) influence of the types of CER with different physical characteristics, described in Table 1; (ii) influence of the amount of time that the AgNPs suspensions are in contact with the resin (0, 15, 30, 60, and 90 min); and (iii) influence of continuous magnetic stirring during the contact time between the resins and the suspension. Additionally, for the AAS measurements, UV-Vis spectrophotometric monitoring was performed to determine possible modifications at the characteristic AgNPs surface plasmon resonance band (SPR band), which could indicate physical-chemical alterations in the nanomaterials due to the contact with the CER.

#### 3.1.1. Dowex 50W-X8 50–100 Mesh

For the optimization study of the parameters influencing the separation capacity of the resin Dowex 50W-X8 50–100 mesh for Ag ion removal from a starch-AgNPs suspension, two separate groups of assays were planned: (i) without stirring, S-OFF, and (ii) S-ON, with stirring ON during the adsorption procedure. Stirring influences the kinetics between the adsorbent and adsorbate by increasing the frequency of the contact between both; hence, it is expected to be a parameter to influence optimization. Considering that previous assays revealed no significant difference between 0.50 or 1.00 g of resin, the present studies were conducted using lower masses of resin, specifically resin masses of 0.25 g. Thus, for each group, (i) and (ii), 0.25 g and 0.50 g of the resin were put in contact with 20 mL of a starch-AgNPs suspension for a total time of 90 min, during which some supernatant aliquots (2 mL) were collected for later UV-Vis and AAS analyses at the time intervals of 15, 30, 60, and 90 min. The obtained results were compiled in Tables S1 and S2. The corresponding purification ratios (%) were calculated and compiled in Table 3.

The experimental results in Table 3 reveal the general increase in the purification ratios with the adsorption time. When using continuous stirring (S-ON) in particular, independently of the mass of resin and of time, a strong positive effect on the purification rate by adsorption was observed. When using S-ON, a rapid increase in the purification ratios before 15 min of contact time can be observed, achieving stabilization for longer contact times. Additionally, despite using S-ON or S-OFF, the use of 0.50 g of resin allowed higher purification ratios to be achieved compared to when 0.25 g of resin was used. The results in Table 3 indicate that using continuous automatic stirring and 0.50 g of CER had a positive influence on the adsorption rates, yielding higher purification ratios.

**Figure 2:**
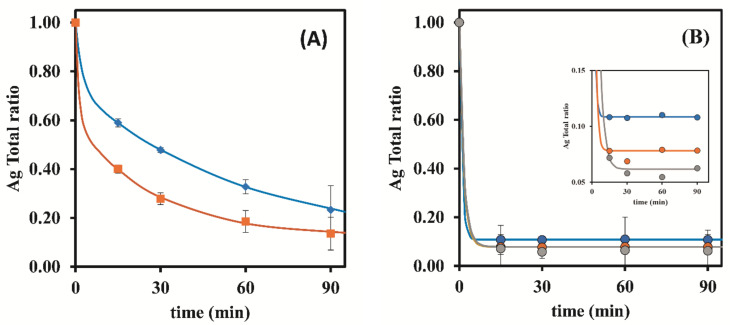
Influence of stirring and resin mass (Dowex 50W-X8 50–100 mesh) on the Ag Total ratio. (**A**)—S-OFF; (**B**)—S-ON (●—0.25 g of resin; ●—0.50 g of resin; ●—1.00 g of resin); inset in (**B**)—amplification of results in the Figure 2B comprising Ag Total ratios till the value of 0.15. The represented values are the average of three replicates for each different assay.

To better evaluate the influence of the studied parameters in the rate at which Ag could be removed from the raw AgNPs suspensions, graphical representations of the Ag Total ratios vs. time are depicted in Figure 2.These graphical representations confirm that by using S-ON (Figure 2B), Ag was able to be removed at higher rates and at faster kinetic rate than without stirring (S-OFF: Figure 2A), regardless of the mass of the resin used.

**Figure 3:**
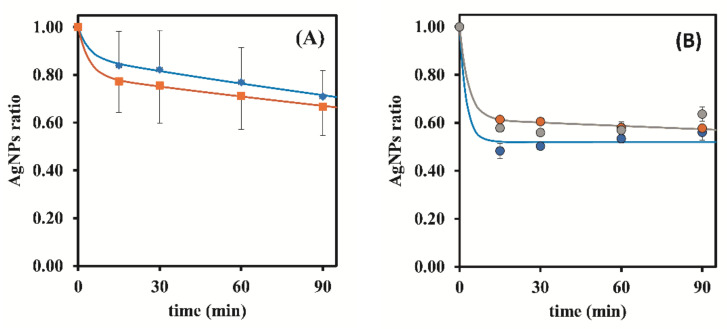
Influence of stirring and resin mass (Dowex 50W-X8 50–100 mesh) on the AgNPs ratio. (**A**)—S-OFF; (**B**)—S-ON (●—0.25 g of resin; ●—0.50 g of resin; ●—1.00 g of resin). The represented values are the average of 3 replicates for each different assay.

**Figure 4:**
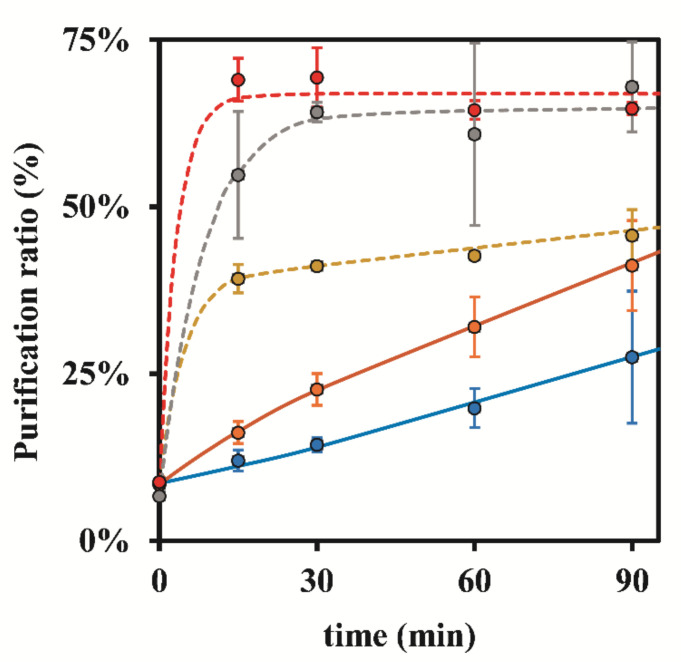
Influence of stirring and resin mass (Dowex 50W-X8 50–100 mesh) on the purification ratio (%) of AgNPs suspensions. ●—0.25 g of resin, S-OFF; ●—0.50 g of resin, S-OFF; ●—0.25 g of resin, S-ON; ●—0.50 g of resin, S-ON; ●—1.00 g of resin, S-ON. The represented values are the average of three replicates for each different assay.

Additionally, the same results revealed that in the absence of stirring, total Ag removal was faster when using 0.50 g of resin, instead of 0.25 g (Figure 2A). In fact, with S-ON, the resin mass did not have a significant influence on the Ag Total ratios (Figure 2B). During that same data analysis but while focusing on the AgNPs ratios related to unwanted AgNPs removal (Figure 3), it was verified that with S-ON (Figure 3B) the AgNPs removal was faster and took place to a higher extent than during S-OFF (Figure 3A). For S-OFF, the use of 0.50 g of resin resulted in a AgNPs removal that was only slightly faster than that achieved when using 0.25 g of resin (Figure 3A), whilst for S-ON (Figure 3B), it was not possible to observe the influence of the amount of resin (0.25 and 0.50 g) on the AgNPs removal rate. Overall, it was revealed that the use of continuous stirring during the adsorption process had a sustained influence, markedly increasing the kinetic indicators of the rates and ratios as well as the purification ratio (%) (Figure 4) despite the AgNPs losses during the adsorption process.

Considering the positive influence of the resin mass on the total Ag removal from the suspensions, one could hypothesize that by further increasing the ratio mass of resin/volume of the suspension in conjunction with S-ON could result in the further improvement of the adsorption indicators. This way, the previous assays were executed using S-ON and a mass fixed at 1.00 g of the resin. The corresponding results were included in Table 3 and were graphically represented in Figure 2B and Figure 3B, together with the previous results with S-ON for comparison purposes.

According with Figure 2B (inset), by increasing the resin mass to 1.00 g, there was no significant improvement in the total Ag removal rate from the suspensions. Additionally, at 15 min, the Ag Total ratios were already very similar to the asymptote of the graphical representation, corresponding to a state of equilibrium in the adsorption phenomena.

By adjusting the exponential models to the results of Ag Total ratios for S-OFF and 0.25 and 0.50 g of resin, these did not converge to a ratio of 1 at time 0 min, which can be explained by the fact that when the suspension and resin come into contact, there is some uncontrolled agitation, and the removal of the Ag ions happens more quickly at that moment, but following, the Ag ions undergo a slow and limiting diffusion step at the resin surface. A good fit was observed when using a double exponential model, reflecting the initial situation where the Ag Total ratio at time 0 min was 1. Additionally, by prolonging the exponential model to longer times such as 300 min, it was noticed that the results obtained with S-OFF converged at a slow rate to the results obtained using S-ON.

Figure 4 contains all of the results that were obtained from the previous assays, representing the change in the purification ratio (%) as function of time, with and without stirring. After 30 min of contact time, the difference between 0.50 and 1.00 g of resin with stirring was not significant since for longer times, both of the results fluctuated between 60.84–69.36%. This way, the results indicated that a contact time of 15 min was perfectly acceptable to attain good purification ratios when using 0.50 g of resin and S-ON.

This study allows us to draw the conclusion that to purify a AgNPs suspension, the use of 0.50 g of Dowex 50W-X8 (50–100 mesh) resin under continuous agitation (S-ON) allows for a total Ag content of about 70% in the form of AgNPs by removing major amounts of free Ag, indicating a major improvement over the initial 8% of the AgNPs % ratio in the raw suspension (Figure 4). This is perhaps, in addition to repeatability/reproducibility, the best indicator for the use of Dowex 50W-X8 (50–100 mesh) resin with continuous stirring to purify AgNPs suspensions. Additionally, it was possible to verify that the lowest purification ratio (%) at 15 min with 1.00 g of resin was certainly due to experimental variability and not to a real effect related to the lower amount of resin.

#### 3.1.2. Bio-Rad 50W-X8 100–200 Mesh

To further assess the effect of the resin particle size on the purification process of AgNPs suspensions using the resin Bio-Rad 50W-X8 100–200 mesh, the parameters influencing the performance were optimized by following the same methodology described previously for the resin Dowex 50W-X8 50–100 mesh.

Considering the confirmed positive effect of agitation (S-ON) on the mixture suspension and resin during the adsorption process, in this study, 0.25, 0.50, and 1.00 g of the resin was put in contact with 20 mL of the raw AgNPs suspension under continuous magnetic stirring for a total time of 90 min. During the experiment, at pre-determined times *t*, an aliquot of supernatant was removed for analysis without interrupting the stirring. The corresponding detailed results are depicted in Table S3 (Supplementary Materials), and the respective purification ratios (%) are summarized in Table 4.

**Figure 5:**
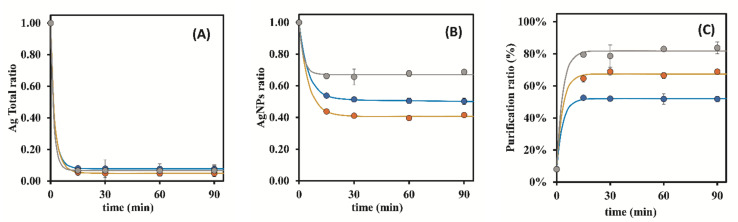
The influence of the resin mass (Bio-Rad 50W-X8 100–200 mesh) on the removal ratio of the Ag^+^ present in suspension over time (●—0.25 g of resin; ●—0.50 g of resin; ●—1.00 g of resin): (**A**)—Ag total ratio in the suspension; (**B**)—AgNPs ratio in the suspension; (**C**)—Purification ratio (%). The represented values are the average of 3 replicates for each different assay.

The graphic in Figure 5A, which represents the variation in ratio of the total amount of Ag (Ag^+^ and AgNPs) in the suspensions with the time, shows an accentuated decrease in that amount up to 15 min of exposure time, and then the Ag levels remain stable up to 90 min, indicating that adsorption achieved an equilibrium state that was independent of the resin mass. This study evidenced the high rate and efficiency of the resin in the removal of ionic silver. Figure 5B shows the influence of the adsorption time on the variation of the AgNPs ratio. By observing the results, one can conclude that the amount of AgNPs decreased rapidly for up to 15 min of adsorption time, stabilizing afterwards until 90 min. Yet, it was also observable different rates varying with the resin mass, and that using 1.00 g of resin generated unexpected results compared to the other masses of 0.25 and 0.50 g, which could be due to some experimental variability. Overall, during the purification process, the AgNPs losses were between approximately 35% and 60%. Figure 5C graphically represents the variation in the purification ratio (%) with the adsorption time. Again, it was observed that the purification ratios stabilized after 15 min of adsorption time. Between 15 min and 90 min of adsorption, the highest average ratio (81.19 ± 0.03%) was attained using 1.00 g of the resin Bio-Rad 50W-X8 100–200 mesh, which was higher than the one obtained when using 1.00 g of the resin Dowex 50W-X8 50–100 mesh (61.13 ± 0.06%).

**Figure 6:**
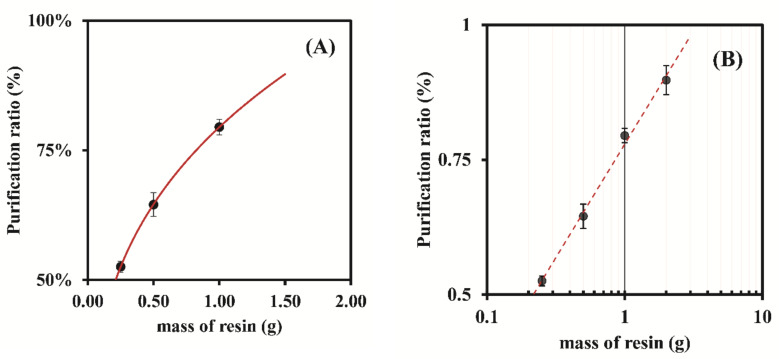
(**A**)—Variation of the Purification ratio (%) at 15 min of adsorption time, with the different masses of resin; (**B**)—Confirmation of the variation of the Purification ratio (%) at 15 min of adsorption time, by varying the resin mass between 0.25–2.00 g (logarithmic scale for the resin mass).

According to the results, a contact time of 15 min and 1.00 g of resin were the optimal conditions obtained for the resin Bio-Rad 50W-X8 100–200 mesh. By graphically representing the influence of the resin mass on the purification ratio % (Figure 6A), one could hypothesize that by using a mass of resin higher than 1.00 g would allow higher purification ratios to be achieved while at the same time assuring the integrity of the majority of AgNPs in the raw suspensions. By fixing the contact time at 15 min, a study was conducted to assess the influence of the resin masses (0.25–2.00 g) of the Bio-Rad 50W-X8 100–200 mesh and the obtained results are represented in Figure 6B. The purification ratio % of the AgNPs obtained for 2.00 g of resin was approximately 90%, higher than the about 81% obtained for 1.00 g of resin. Thus, according with the results, it could be possible to purify raw AgNPs suspensions starting at roughly 8% to 90% (90% of the total silver ion the purified suspension is in the reduced form of AgNPs) using 2.00 g of the resin Bio-Rad 50W-X8 100–200 mesh, an adsorption time of 15 min, and continuous automatic stirring.

#### 3.1.3. Bio-Rad 50W-X8 200–400 Mesh

Following the studies of the influence of the cationic resin characteristics on the Ag adsorption from the raw AgNPs suspensions an assay using 0.25, 0.50, and 1.00 g of the resin Bio-Rad 50W-X8 200–400 mesh was conducted under continuous magnetic stirring. In Table S4 (Supplementary Materials) the results of the UV-Vis and AAS analyses are compiled. The respective purification ratios (%) are summarized in Table 5.

**Figure 7:**
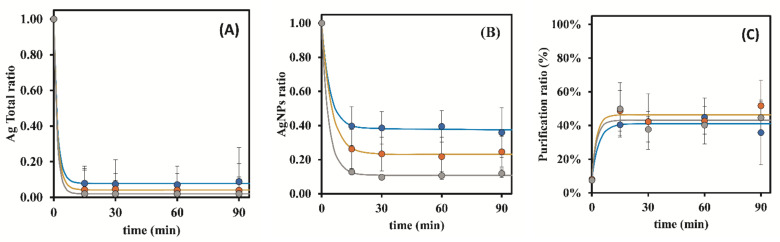
Influence of the resin mass (Bio-Rad 50W-X8 200–400 mesh) on the removal ratio of Ag^+^ present in suspension over time (●—0.25 g of resin; ●—0.50 g of resin; ●—1.00 g of resin): (**A**)—Ag total ratio in the suspension; (**B**)—AgNPs ratio in the suspension; (**C**)—Purification ratio (%). The represented values are the average of 3 replicates for each different assay.

The assay results revealed that after 15 min of contact time, there was a rapid decrease (about 90%) in the amount of total silver in the suspension (Figure 7A). Overall, this resin (Bio-Rad 50W-X8 200–400 mesh) revealed a higher capacity to adsorb Ag, free Ag, and Ag in the form of AgNPs, meaning that some of these nanoparticles disintegrated during the purification process (Figure 7B). For this reason, the verified amount of AgNPs present in the nanoparticles was very low after the purification process (diminishing between 60–90%, at 15 min of contact time) compared to the other tested resins. This justifies the observation of significant lower purification ratios in Figure 7C, namely between 40% and 50% at 15 min of contact time, compared to the other studied resins (Dowex 50W-X8 50–100 mesh: 40–70%; Bio-Rad 50W-X8 100–200 mesh: 50–80%, at 15 min of contact time).

**Figure 8:**
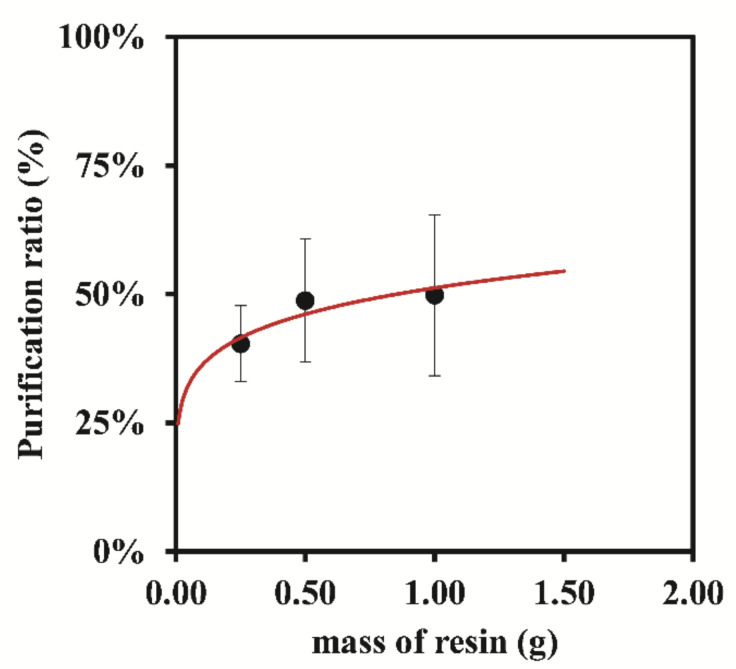
Variation in the purification ratio (%) at 15 min of adsorption time with the different masses of the resin Bio-Rad 50W-X8 200–400 mesh.

Following this, a study was conducted to evaluate the influence of the resin mass in the purification ratio (%) by keeping constant the contact time at 15 min, and the obtained results are represented in Figure 8. The mass of the resin used has an impact on the velocity at which the Ag ions are removed from the samples. For small resin/metal ratios, the resin capacity, expressed as the mass of ions removed per unit of mass of resin, is kept fairly constant. Further increase of the resin mass/ions ratio will eventually lead to a plateau where the amount of metal ions adsorbed is not dependent on the resin mass, (the apparent resin capacity will drop) particularly when the resin is allowed to contact with the sample enough time to reach equilibrium. In the current experiments, it was observed that equilibrium was reached before the first time point (15 min), see Figure 7. The concentration of Ag ions at equilibrium, both free Ag and AgNPs, lowers as the resin mass increases but the ratio of AgNPs/Total Ag shows no significant change when the resin mass is increased from 0.50 g to 1.00 g.

Considering the results from the studies determining the influence of using three resin types and that mainly focused on different particle sizes, it was concluded that the best compromise between the removal of Ag free ions and the maintenance of initial AgNPs concentration and integrity during Ag adsorption from raw AgNPs suspensions was achieved when using 0.50 g of the resin Bio-Rad 50W-X8 100–200 mesh and a contact time of 15 min.

According with the manufacturer, the cation exchange resin Bio-Rad 50W-X8 is stable in acid, base, and organic solvents, and it can also be autoclaved to resolve potential problems arising from bacterial growth during storage. Additionally, its regeneration is easily achieved by washing the resin with a solution of a strong acid, such as hydrochloric acid or nitric acid.

## 4. Conclusions

In this work, the use of cationic exchange resins to separate free silver ions in suspensions of synthetized silver nanoparticles was evaluated. These free ions are the by-products originated from the AgNPs synthesis procedure due to Ag free ions not being reduced to the fundamental states that are the building blocks of the nanomaterials. Since silver ions are chemically reactive and possess the strong capacity to severely interfere with biological processes, they are often used in pharmaceutical formulations of antiseptics or wound bandages, and the presence of these ions prevents accurate results from being obtained in biological assays, namely in antimicrobial, antifungal, and anticancer assays. Silver ions can even interfere with the methodologies used in biochemical assays. Aiming to achieve the best compromise between the yield of AgNPs synthesis enabled by green approaches and their loss throughout the purification process, this work revealed that the use of 2.00 g of the cationic resin Bio-Rad 50W-X8 with particle size 100–200 mesh resulted in purification ratios of about 90% with only 15 min of contact time with the raw suspension, when the initial suspension had only 8% of the amount of AgNPs in relation to the total quantified Ag. This means that there was an improvement in the raw AgNPs suspension regarding the presence of silver ions of about 10 times, which is definitely a significant result. The proposed methodology is low cost, easy to perform, expeditious, and environmentally friendly, while providing a simple approach that is able to diminish the amount of free silver ions in AgNPs suspensions to a high extent at the same time.

## Figures and Tables

**Table 1:** Properties of the studied cationic exchange resins.

Resin.	Matrix	Particle Size (Mesh)	Cross-Linkage	Capacity (meq/mL)
Dowex 50W-X8	styrene divinylbenzene	50–100	8%	1.1
Bio-Rad 50W-X8	styrene divinylbenzene	100–200	8%	1.7
Bio-Rad 50W-X8	styrene divinylbenzene	200–400	8%	1.7

**Table 2:** Preliminary results for AgNPs purification with the resin Dowex 50W-X8 50–100 mesh, for resin masses of 0.50 and 1.00 g.

Contact Time (h)	0.50 g		1.00 g	
	C_Ag total_ (mg/L)	C_Ag in AgNPs_ (mg/L)	C_Ag total_ (mg/L)	C_Ag in AgNPs_ (mg/L)
**0**	1176.80	66.94	959.20	69.97
**1**	90.09	38.84	121.59	58.50
**2**	45.56	40.34	68.03	63.16

**Table 3:** Purification ratios (%) obtained for Dowex 50W-X8 resin 50–100 mesh (results correspond to the average of three replicates ± standard deviation).

	Resin Mass (g)	Time of Contact (min)				
		0	15	30	60	90
S-OFF	0.25	8.44 ± 1.18	12.01 ± 1.60	14.36 ± 1.05	19.85 ± 2.91	27.49 ± 9.91
	0.50	8.44 ± 1.18	16.20 ± 1.66	22.66 ± 2.37	32.00 ± 4.48	41.21 ± 6.72
S-ON	0.25	8.80 ± 0.14	39.25 ± 2.11	41.12 ± 0.29	42.65 ± 0.30	45.70 ± 3.86
	0.50	8.80 ± 0.14	69.02 ± 3.20	69.36 ± 4.43	64.49 ± 1.39	64.68 ± 0.93
	1.00	6.68 ± 0.03	54.75 ± 9.48	64.16 ± 1.46	60.84 ± 13.64	67.95 ± 6.72

S-OFF: stirring off; S-ON: stirring on.

**Table 4:** Purification ratios (%) obtained for Bio-Rad 50W-X8 100–200 mesh (results correspond to the average of three replicates ± standard deviation).

Resin Mass (g)	Time of Contact (min)				
	0	15	30	60	90
0.25	7.90 ± 0.06	52.55 ± 1.07	51.96 ± 1.60	51.71 ± 2.38	51.74 ± 2.27
0.50	8.05 ± 0.05	64.55 ± 2.02	68.91 ± 2.43	66.48 ± 1.74	68.81 ± 0.54
1.00	8.01 ± 0.10	79.48 ± 1.81	78.58 ± 6.48	82.98 ± 2.05	83.62 ± 2.80

**Table 5:** Purification ratios (%) obtained for Bio-Rad 50W-X8 200–400 mesh (results correspond to the average of 3 replicates ± standard deviation).

Resin Mass (g)	Time of Contact (min)				
	0	15	30	60	90
0.25	8.10 ± 0.92	40.39 ± 7.37	42.39 ± 6.09	44.92 ± 6.27	35.80 ± 19.18
0.50	8.10 ± 0.92	48.75 ± 11.94	42.30 ± 16.50	42.62 ± 13.55	51.74 ± 15.00
1.00	7.50 ± 1.00	49.80 ± 15.62	37.72 ± 7.45	40.26 ± 5.30	44.66 ± 9.45

## Data Availability

Data can be available upon request from the authors.

## Supplementary Materials

The following supporting information can be downloaded at: https://www.mdpi.com/article/10.3390/nano12040644/s1, Table S1: UV-Vis data and AAS results of Dowex 50W-X8 resin (0.25 and 0.50 g) with S-OFF (time of adsorption 0–90 min); Table S2: UV-Vis data and AAS results of Dowex 50W-X8 resin (0.25, 0.50 and 1.00 g) with S-ON (time of adsorption 0–90 min); Table S3: UV-Vis data and AAS results of Bio-Rad 50W-X8 100–200 mesh resin (0.25, 0.50 and 1.00 g) with S-ON (time of adsorption 0–90 min); Table S4: UV-Vis data and AAS results of Bio-Rad 50W-X8 resin: 200–400 mesh (0.25, 0.50 and 1.00 g) with S-ON (time of adsorption 0–90 min).
