# Supplementary material for: Minimizing the Silver Free Ion Content in Starch Coated Silver Nanoparticle Suspensions with Exchange Cationic Resins
Source: Nanomaterials (Basel). 2022 Feb 15;12(4):644. doi: 10.3390/nano12040644 (PMC8877803; doi:10.3390/nano12040644)
Supplement: Supplementary file 1 [file nanomaterials-12-00644-s001.zip › nanomaterials-1569635-supplementary.pdf]

Supplementary Materials

Minimizing the Silver Free Ion Content in Starch Coated Silver Nanoparticle Suspensions with Exchange Cationic Resins

Catarina S. M. Martins [1], Alberto N. Araújo [1], Luís Pleno de Gouveia [2,*] and João A. V. Prior [1,*]

[1] LAQV, REQUIMTE, Laboratory of Applied Chemistry, Department of Chemical Sciences, Faculty of Pharmacy, University of Porto, 4050-313 Porto, Portugal
[2] Pharmacological and Regulatory Sciences Group (PharmaRegSci), Research Institute for Medicines (iMed.ULisboa), Faculdade de Farmácia da Universidade de Lisboa, Lisbon, 1649-003, Portugal
* Correspondence: lgouveia@campus.ul.pt (L.P.G.); joaoavp@ff.up.pt (J.A.V.P.)

Citation: Martins, C.S.M.; Araújo, A.N.; de Gouveia, L.P.; Prior, J.A.V. Minimizing the Silver Free Ion Content in Starch Coated Silver Nanoparticle Suspensions with Exchange Cationic Resins. *Nanomaterials* **2022**, *12*, 644. https://doi.org/10.3390/nano12040644

Academic Editor: Tai-Chia Chiu

Received: 8 January 2022
Accepted: 7 February 2022
Published: 15 February 2022

Publisher's Note: MDPI stays neutral with regard to jurisdictional claims in published maps and institutional affiliations.

Copyright: © 2022 by the authors. Submitted for possible open access publication under the terms and conditions of the Creative Commons Attribution (CC BY) license (https://creativecommons.org/licenses/by/4.0/).

Table S1. UV-Vis data and AAS results of Dowex 50W-X8 resin (0.25 and 0.50 g) with S-OFF (time of adsorption 0–90 min).

		0.25 g			0.50 g		
**Time (min)**	**Samples**	**λ_{Max}**	**Abs_{Max}**	**C_{Ag+} (mg/L)**	**λ_{Max}**	**Abs_{Max}**	**C_{Ag+} (mg/L)**
**0**	S_0	408.2	0.824	1040.8	408.2	0.824	1040.8
	S_0	405.8	0.910	984.4	405.8	0.910	984.4
	S_0	408.2	0.663	1001.2	408.2	0.663	1001.2
	RS_0	411.2	0.714	100.9	411.2	0.714	100.9
	RS_0	408.8	0.839	81.8	408.8	0.839	81.8
	RS_0	409.6	0.692	73.4	409.6	0.692	73.4
**15**	S_{15}	409.0	0.734	501.4	408.2	0.765	348.4
	S_{15}	407.8	0.828	639.8	406.8	0.858	437.2
	S_{15}	408.4	0.749	636.4	407.8	0.739	423.4
	RS_{15}	409.4	0.632	68.2	408.2	0.628	63.1
	RS_{15}	408.6	0.778	76.8	408.4	0.772	67.4
	RS_{15}	409.6	0.650	66.3	409.2	0.674	63.9
**30**	S_{30}	408.8	0.640	413.3	408.2	0.760	252.3
	S_{30}	407.4	0.798	517.6	406.8	0.866	263.4
	S_{30}	408.0	0.736	512.0	407.8	0.690	326.6
	RS_{30}	409.2	0.637	64.1	409.4	0.629	58.8
	RS_{30}	408.8	0.756	73.2	408.0	0.739	64.9
	RS_{30}	410.2	0.637	68.8	409.0	0.642	65.4
**60**	S_{60}	408.4	0.736	266.7	408.2	0.763	181.1
	S_{60}	407.2	0.822	344.9	406.6	0.867	172.1
	S_{60}	409.0	0.787	377.1	407.6	0.733	209.3
	RS_{60}	409.0	0.587	61.6	408.2	0.575	55.6
	RS_{60}	408.4	0.660	65.5	407.6	0.592	63.6
	RS_{60}	411.6	0.559	65.9	409.0	0.641	59.2
**90**	S_{90}	408.2	0.748	152.1	408.0	0.775	153.5
	S_{90}	407.2	0.846	275.6	406.6	0.879	120.9
	S_{90}	407.4	0.780	273.6	407.4	0.684	137.3
	RS_{90}	408.0	0.561	59.1	408.0	0.563	53.5
	RS_{90}	408.0	0.666	63.2	405.6	0.564	58.3
	RS_{90}	409.8	0.565	56.6	409.0	0.603	55.7

S: AgNPs suspension before precipitation method; RS: AgNPs suspension after the precipitation method; λ_{Max}: maximum wavelength; Abs_{Max}: maximum absorbance.

Table S2. UV-Vis data and AAS results of Dowex 50W-X8 resin (0.25, 0.50 and 1.00 g) with S-ON (time of adsorption 0–90 min).

		0.25 g resin			0.50 g resin			1.00 g resin		
**Time (min)**	**Samples**	**λ_{Max}**	**Abs_{Max}**	**$[Ag^+]$ (mg/L)**	**λ_{Max}**	**Abs_{Max}**	**$[Ag^+]$ (mg/L)**	**λ_{Max}**	**Abs_{Max}**	**$[Ag^+]$ (mg/L)**
**0**	S_0	407.2	0.963	1103.2	407.2	0.963	1103.20	407.2	0.830	1250.8
	S_0	407.2	0.963	1094.8	407.2	0.963	1094.80	407.2	0.830	1251.2
	S_0	407.2	0.963	1067.6	407.2	0.963	1067.60	407.2	0.830	1245.2
	RS_0	411.4	0.615	96.3	411.4	0.615	96.34	409.2	0.629	83.4
	RS_0	411.4	0.615	95.2	411.4	0.615	95.22	409.2	0.629	84.0
	RS_0	411.4	0.615	95.7	411.4	0.615	95.69	409.2	0.629	82.9
**15**	S_{15}	407.6	0.704	119.2	406.8	0.758	83.28	407.2	0.713	102.4
	S_{15}	408.4	0.737	118.2	407.0	0.761	87.61	407.0	0.678	94.6
	S_{15}	408.8	0.727	116.0	406.8	0.790	84.63	407.0	0.731	72.7
	RS_{15}	409.2	0.413	45.9	406.2	0.686	60.55	403.8	0.479	48.8
	RS_{15}	409.0	0.412	49.2	406.4	0.663	58.70	404.0	0.518	48.3
	RS_{15}	409.2	0.362	43.6	405.4	0.608	57.00	404.0	0.502	47.6
**30**	S_{30}	408.8	0.719	118.6	407.0	0.761	84.88	407.0	0.707	71.4
	S_{30}	408.6	0.741	118.6	407.0	0.787	86.05	407.4	0.673	73.4
	S_{30}	407.8	0.738	114.2	407.0	0.781	80.00	406.8	0.734	73.5
	RS_{30}	409.8	0.460	49.1	406.4	0.610	57.15	404.2	0.492	46.1
	RS_{30}	409.2	0.416	48.4	405.4	0.644	57.05	405.4	0.523	48.0
	RS_{30}	409.8	0.350	47.0	404.8	0.658	59.55	404.6	0.535	46.0
**60**	S_{60}	408.0	0.756	115.9	407.0	0.752	85.60	407.0	0.737	69.6
	S_{60}	408.2	0.765	122.8	407.0	0.785	82.80	407.4	0.690	72.3
	S_{60}	407.8	0.777	121.1	407.0	0.804	90.00	407.0	0.762	99.7
	RS_{60}	409.2	0.499	49.8	407.0	0.618	54.53	405.4	0.535	50.8
	RS_{60}	410.0	0.456	52.4	406.4	0.595	54.73	404.6	0.495	45.9
	RS_{60}	408.8	0.430	51.3	407.2	0.610	57.30	405.2	0.536	46.0
**90**	S_{90}	408.4	0.725	114.9	407.0	0.774	84.59	407.0	0.746	84.5
	S_{90}	408.2	0.744	120.1	407.2	0.784	88.05	407.2	0.708	75.6
	S_{90}	408.6	0.748	117.8	407.0	0.812	83.83	406.8	0.784	75.3
	RS_{90}	408.2	0.562	56.8	408.2	0.562	55.50	405.4	0.523	51.7
	RS_{90}	407.2	0.611	50.1	407.2	0.611	56.13	404.4	0.476	56.4
	RS_{90}	407.6	0.644	54.1	407.2	0.596	54.23	404.4	0.471	51.2

S: AgNPs suspension before precipitation method; RS: AgNPs suspension after the precipitation method; λ_{Max}: maximum wavelength; Abs_{Max}: maximum absorbance.

Table S3. UV-Vis data and AAS results of Bio-Rad 50W-X8 100-200 mesh resin (0.25, 0.50 and 1.00 g) with S-ON (time of adsorption 0–90 min).

		0.25 g resin			0.50 g resin			1.00 g resin		
**Time (min)**	**Samples**	**λ_{Max}**	**Abs_{Max}**	**$[Ag^+]$ (mg/L)**	**λ_{Max}**	**Abs_{Max}**	**$[Ag^+]$ (mg/L)**	**λ_{Max}**	**Abs_{Max}**	**$[Ag^+]$ (mg/L)**
**0**	S_0	406.6	0.897	1038.8	406.4	0.892	1094.4	407.0	0.885	1032.4
	S_0	406.6	0.897	1032.4	406.4	0.892	1091.6	407.0	0.885	1030.8
	S_0	406.6	0.897	1032.4	406.4	0.892	1101.6	407.2	0.885	1025.6
	RS_0	409.8	0.637	81.4	407.8	0.654	88.4	409.2	0.620	82.3
	RS_0	409.8	0.637	81.8	407.8	0.654	88.7	409.2	0.620	83.7
	RS_0	409.8	0.637	82.1	407.8	0.654	88.4	409.2	0.620	81.3
**15**	S_{15}	409.2	0.517	83.2	407.8	0.374	58.9	407.2	0.749	68.3
	S_{15}	410.2	0.478	84.4	407.8	0.369	59.9	407.6	0.751	67.7
	S_{15}	410.2	0.503	83.5	407.8	0.377	61.4	406.4	0.803	69.6
	RS_{15}	409.0	0.492	44.4	405.0	0.377	39.5	403.0	0.490	53.7
	RS_{15}	409.0	0.439	44.5	405.6	0.380	37.5	403.0	0.506	54.9
	RS_{15}	409.2	0.417	43.0	405.0	0.346	39.3	402.2	0.527	54.9
**30**	S_{30}	410.0	0.503	80.4	408.0	0.383	52.7	406.8	0.751	68.2
	S_{30}	409.8	0.493	80.7	407.6	0.401	53.0	406.4	0.840	69.5
	S_{30}	410.4	0.481	81.6	407.4	0.400	52.8	407.0	0.804	68.7
	RS_{30}	409.2	0.446	40.9	404.6	0.314	35.1	403.0	0.524	57.6
	RS_{30}	409.8	0.436	42.2	405.0	0.346	36.2	402.8	0.503	55.8
	RS_{30}	409.6	0.436	43.0	404.8	0.344	37.9	403.0	0.490	48.8
**60**	S_{60}	410.4	0.473	81.6	408.0	0.372	52.5	407.0	0.782	66.7
	S_{60}	410.4	0.475	77.5	407.6	0.401	52.6	407.2	0.792	67.0
	S_{60}	410.4	0.440	80.4	407.4	0.359	53.1	406.8	0.837	68.4
	RS_{60}	409.8	0.394	39.8	407.4	0.355	35.7	402.8	0.520	55.8
	RS_{60}	409.6	0.409	42.8	406.8	0.355	33.9	402.2	0.511	55.1
	RS_{60}	410.0	0.403	41.2	406.8	0.352	35.6	402.4	0.554	56.8
**90**	S_{90}	410.4	0.448	79.9	407.8	0.343	53.1	407.0	0.828	66.3
	S_{90}	411.0	0.451	80.1	407.6	0.372	53.6	407.2	0.860	68.1
	S_{90}	410.4	0.432	78.2	407.8	0.398	53.5	406.2	0.868	69.0
	RS_{90}	409.4	0.401	39.9	405.6	0.367	36.5	402.8	0.563	57.6
	RS_{90}	409.2	0.416	42.9	407.0	0.403	36.7	402.0	0.561	57.5
	RS_{90}	407.6	0.644	40.4	406.8	0.380	37.0	402.6	0.501	54.9

S: AgNPs suspension before precipitation method; RS: AgNPs suspension after the precipitation method; λ_{Max}: maximum wavelength; Abs_{Max}: maximum absorbance.

Table S4. UV-Vis data and AAS results of Bio-Rad 50W-X8 resin: 200-400 mesh (0.25, 0.50 and 1.00 g) with S-ON (time of adsorption 0–90 min).

		**0.25 g resin**			**0.5 g resin**			**1.0 g resin**		
**Time (min)**	**Samples**	**λ_{Max}**	**Abs_{Max}**	**$[Ag^+]$ (mg/L)**	**λ_{Max}**	**Abs_{Max}**	**$[Ag^+]$ (mg/L)**	**λ_{Max}**	**Abs_{Max}**	**$[Ag^+]$ (mg/L)**
**0**	S_0	407.4	0.804	1118.0	407.4	0.804	1118.0	407.4	0.804	1118.0
	S_0	406.6	0.851	1123.6	406.6	0.851	1123.6	406.6	0.851	1123.6
	S_0	408.8	0.956	1054.4	408.8	0.956	1054.4	409.4	0.750	1073.2
	RS_0	409.0	0.610	96.7	409.0	0.610	96.7	409.0	0.610	96.7
	RS_0	407.6	0.703	79.0	407.6	0.703	79.0	407.6	0.703	79.0
	RS_0	409.0	0.738	90.8	409.0	0.738	90.8	410.8	0.533	73.1
**15**	S_{15}	411.4	0.229	80.9	411.6	0.139	38.8	411.6	0.098	20.9
	S_{15}	408.8	0.338	80.9	408.8	0.166	43.1	408.8	0.084	22.3
	S_{15}	410.0	0.531	95.8	410.0	0.435	56.8	412.4	0.083	22.2
	RS_{15}	409.6	0.265	27.4	408.8	0.155	16.3	408.6	0.128	14.1
	RS_{15}	408.8	0.349	31.5	408.8	0.149	17.9	403.6	0.070	9.8
	RS_{15}	408.8	0.524	46.4	408.8	0.410	35.5	412.2	0.130	8.4
**30**	S_{30}	410.6	0.222	69.5	412.6	0.113	60.1	411.2	0.087	23.5
	S_{30}	408.8	0.281	77.5	409.0	0.133	36.5	408.8	0.060	19.3
	S_{30}	410.2	0.513	93.8	410.0	0.397	52.9	412.2	0.084	21.5
	RS_{30}	409.0	0.310	30.2	409.8	0.132	16.0	409.8	0.108	10.8
	RS_{30}	409.0	0.299	27.8	407.6	0.114	14.8	405.2	0.080	6.8
	RS_{30}	408.8	0.504	44.9	409.2	0.376	31.5	426.0	0.190	6.9
**60**	S_{60}	411.4	0.182	69.6	411.4	0.084	39.2	410.2	0.078	25.8
	S_{60}	408.4	0.267	74.7	408.4	0.112	36.7	408.8	0.045	19.2
	S_{60}	409.8	0.515	87.9	410.2	0.395	54.7	438.8	0.221	20.9
	RS_{60}	411.2	0.234	31.0	411.6	0.128	12.1	412.2	0.049	10.9
	RS_{60}	409.2	0.283	29.0	408.8	0.117	14.5	408.8	0.143	6.6
	RS_{60}	408.8	0.509	45.2	408.6	0.382	31.4	438.8	0.221	9.2
**90**	S_{90}	412.0	0.149	129.5	411.6	0.082	35.0	410.4	0.091	23.5
	S_{90}	408.8	0.238	76.3	408.8	0.105	37.4	408.4	0.067	19.6
	S_{90}	409.4	0.508	84.2	410.0	0.357	55.1	412.2	0.064	23.3
	RS_{90}	410.6	0.174	21.1	410.0	0.104	22.3	406.0	0.089	13.0
	RS_{90}	408.8	0.269	27.9	407.2	0.105	13.1	404.4	0.112	7.2
	RS_{90}	409.0	0.519	46.0	408.0	0.362	31.2	409.6	0.070	9.8

S: AgNPs suspension before precipitation method; RS: AgNPs suspension after the precipitation method; λ_{Max}: maximum wavelength; Abs_{Max}: maximum absorbance.
